# Anemia in Pregnancy: Effects on Maternal and Neonatal Outcomes at a University Hospital in Riyadh

**DOI:** 10.7759/cureus.27238

**Published:** 2022-07-25

**Authors:** Ibtihal A Bukhari, Najla M Alzahrani, Gharam A Alanazi, Maram A Al-Taleb, Hawail S AlOtaibi

**Affiliations:** 1 Obstetrics and Gynaecology, King Abdullah bin Abdulaziz University Hospital, Princess Nourah bint Abdulrahman University, Riyadh, SAU; 2 Obstetrics and Gynaecology, Princess Nourah bint Abdulrahman University, Riyadh, SAU

**Keywords:** pregnancy complications, congenital malformations, postpartum hemorrhage, low birthweight, anemia

## Abstract

Background: Anemia in pregnancy has a number of adverse effects. This study aims to estimate anemia prevalence in pregnant women and examine the associations between maternal anemia with maternal characteristics, maternal outcomes during pregnancy and delivery, and neonatal outcomes at a university hospital in Riyadh.

Materials and methods: A cross-sectional study was undertaken among 400 women who delivered at the hospital. Data were collected through a data extraction sheet. Multivariate analysis was adopted according to the results of univariate analysis.

Results: Overall anemia prevalence was 39% (including 21% moderate anemia and 18% mild anemia); the rest, 61%, were normal. Non-intake of intravenous iron was more common among mothers with mild anemia (65.3%) compared to normal and moderately anemic (p=0.001). Significant differences between groups were found in relation to maternal outcomes such as pregnancy-induced hypertension (p=0.019), antepartum hemorrhage (p=0.001), postpartum hemorrhage (p=0.002), and non-intake of blood transfusion during pregnancy (p=0.012) and emergency cesarean section (p=0.017). Neonatal outcomes, including congenital malformations (p=0.003) and admission to the neonatal intensive care unit (NICU) (p<0.001), were higher in mildly anemic mothers. Statistically significant relationships were found between anemia in pregnancy and postpartum hemorrhage (odds ratio [OR] = 3.61; confidence interval [CI] 1.52-8.58; p=0.004), congenital malformations (OR = 5.09; CI 1.81-14.29; p=0.002), NICU admissions (OR=8.32; CI 2.77-24.96; p=0.001), and low birth weight (LBW; OR=1.833; CI 1.021-3.294; p=0.042).

Conclusions: The study highlights the association of maternal anemia with adverse events in mothers, such as postpartum hemorrhage. Among neonates, congenital malformations, low birth weight, and higher admissions to the NICU have been reported.

## Introduction

Anemia, a condition characterized by reduced concentrations of hemoglobin and decreased oxygen-carrying capacity of the blood, is globally considered a serious public health issue [1]. Women of reproductive age are particularly vulnerable since the World Health Organization (WHO) estimates one-third of this group to be anemic [2]. There are different types of anemia depending on the root cause. For instance, nutritional anemia arises due to the deficiency of iron, vitamin A, vitamin B12, folate, and riboflavin, while certain anemias, such as sickle cell anemia and thalassemia, are hereditary [3].

Anemia during pregnancy is a very common complication that affects 40% of pregnant women worldwide [2], and in Saudi Arabia, the prevalence was estimated at 27.3% in 2019 [4]. According to the World Health Organization (WHO), pregnant women are considered anemic when their hemoglobin levels are less than 11 g/dl [5]. 

Given the high prevalence, anemia during pregnancy has been widely investigated to examine its impact on maternal and fetal outcomes, and it was reported that severe anemia during pregnancy could have significant adverse effects on the mother and the baby, and hemoglobin levels of less than 6 g/dl are associated with poor pregnancy outcomes [1]. A study conducted by Kavle et al. in Tanzania reported a strong relationship between the presence of moderate-to-severe maternal anemia at 28 weeks of gestation and the increased severity of blood loss during childbirth and the first 24 hours postpartum [6]. Another study carried out by Kumar et al. in India reported maternal anemia to be associated with adverse fetal outcomes when compared to non-anemic mothers, including a 6.5% increase in the incidence of low birth weight (LBW) babies and an 11.5% increase in the incidence of preterm deliveries if the mother is anemic in her third trimester [7]. Meanwhile, Stephan et al. highlighted the risk factors of maternal anemia as being multifactorial; these factors include iron, folate, vitamin A and B12 deficiencies and infections such as malaria, hookworm, tuberculosis, and human immuno-deficiency virus [8].

Conversely, there have been studies that have reported no association between maternal anemia and adverse pregnancy outcomes [9-11]. Moreover, Malhotra et al. implied mild anemia to confer a protective role against LBW [12]. Thus, despite the growing number of studies on this topic, there has been a general lack of consensus on the effect of maternal anemia on pregnancy outcomes.

Therefore, this study aims to estimate the prevalence of anemia in pregnant women, to examine the association between maternal anemia status with maternal characteristics, maternal outcomes during pregnancy and delivery, and neonatal outcomes at King Abdullah bin Abdulaziz University Hospital (KAAUH) in Riyadh, Saudi Arabia.

## Materials and methods

The study was approved by the Institutional Review Board of Princess Nourah bint Abdulrahman University with an ethical approval number of 20-0030.

This is a cross-sectional study conducted in the obstetrics/gynecology department at King Abdullah bin Abdulaziz University Hospital (KAAUH). All medical records of pregnant women who gave birth at King Abdullah bin Abdulaziz University Hospital (KAAUH) between 2018 and 2019 were included in the study. Medical records of pregnant women who gave birth before 2018 and after 2019 were excluded from the study.

Sample size calculation

Previous literature reported that the prevalence of anemia among pregnant women in Saudi Arabia was 27.3% in 2019. Based on this, the sample size was calculated using the below formula:

$n = (Z^{2} * p(1-p))/d^{2}$

where *n* is the sample size, *Z* is the level of confidence (two-sided 95% confidence interval), and *P* is the prevalence of anemia from the previous study = 27.3% = precision (5%).

The minimal sample size needed for the current study was calculated to be 305, and a total of 400 pregnant women were included in the study. The prevalence of anemia among them was calculated based on the WHO definition of anemia for pregnant women: severe (<7 g/dl), moderate (7 to <10 g/dl), mild (≥10 to <11 g/dl) or normal (≥11 g/dl).

This study collected the data through a data extraction sheet that contained the following sections: (i) sociodemographic characteristics of the patient, including age and nationality; (ii) obstetric history of the mother including gravidity, parity, history of abortion, gestational age at delivery, and date of delivery; (iii) pre-pregnancy and antenatal care including folic acid, ferritin, and vitamin B12 intake; ​​​​​​​(iv) clinical conditions including diabetes, hypertension, renal impairment, and liver diseases; ​​​​​​​(v) mother’s blood level of hemoglobin during and after delivery, HbA1c at booking and third trimester, fasting blood sugar, maternal height, and weight; ​​​​​​​(vi) complications during pregnancy, including preeclampsia, amniotic fluid abnormalities, and intrauterine growth retardation; ​​​​​​​(vii) outcome of pregnancy including mode of delivery, admission to ICU, antepartum and postpartum hemorrhage, need for blood transfusion, and amount of blood loss in case of Cesarean section; ​​​​​​​(viii) neonatal outcomes including living status, birth weight, APGAR score, length of the baby, admission to ICU, preterm or full term, congenital anomalies, and lab work for the cord blood.

Statistical analysis

The minimal sample size needed for the current study was calculated to be 305, and a total of 400 pregnant women were included in the study. The prevalence of anemia among them was calculated based on data analyzed by using the Statistical Package for Social Studies (SPSS 22; IBM Corp., New York, NY, USA). Continuous variables were expressed as mean ± standard deviation and categorical variables were expressed as percentages. A chi-square test was used for categorical variables. Univariate and multivariate logistic regression were used to assess the associated risk factors for anemia in pregnancy. A P-value of <0.05 was considered statistically significant.

## Results

Out of the 400 pregnant women who were included in the study, 96% (n=384) were Saudi and the mean age was 30.59 ± 6.00. Approximately half of the pregnant women’s gravidity was three and above 49% (n=196), while 50.2% (n=201) had a parity of three or more. The overall prevalence of anemia was 39% (n=156) with 21% (n=84) having moderate anemia, 18% (n=72) mild anemia, and 61% (n=244) normal (Table 1).

**Table 1:** Socio-demographic and health characteristics of the pregnant women

Socio-demographic/health characteristics	N (%) or average
Age	30.59 ± 6.00
Nationality	
Saudi	384 (96%)
Non-Saudi	16 (4.0%)
Gravidity	
First pregnancy	91 (22.8%)
Second pregnancy	113 (28.2%)
Third pregnancy and above	196 (49.0%)
Parity	
One child	126 (31.5%)
Two children	73 (18.3%)
Three children and above	201 (50.2%)
Prevalence of anemia	
Moderate (7 to <10 g/dl)	84 (21%)
Mild (≥10 to <11 g/dl)	72 (18%)
Normal (≥11 g/dl)	244 (61%)

Table 2 displays the characteristics of the mothers by their anemia status. With regard to the intake of intravenous iron, more mothers with mild anemia 65.3% (n=47) did not take intravenous iron as compared to 57.1% (n=48) of moderately anemic and 58.6% (n=143) normal mothers, the difference between the groups being statistically significant (p=0.001).

**Table 2:** Maternal characteristics in relation to anemia status *Significant p-value

Maternal characteristics		Maternal hemoglobin g/dl						P-value
		Moderate		Mild		Normal		
		7–9.9 g/dl		10–10.9 g/dl		≥11 g/dl		
		Number	%	Number	%	Number	%	
Was the mother taking folic acid	No	0	0	0	0	2	0.8	0.061
	Yes	78	92.9	62	86.1	231	94.7	
	Not known	6	7.1	10	13.9	11	4.5	
Was the mother taking intravenous iron	No	48	57.1	47	65.3	143	58.6	0.001*
	Yes	12	14.3	2	2.8	7	2.9	
	Not known	24	28.6	23	31.9	94	38.5	
Was the mother taking vitamin B12	No	1	1.2	0	0	2	0.8	0.189
	Yes	76	90.5	61	84.7	226	92.6	
	Not known	7	8.3	11	15.3	16	6.6	
Diabetes status	Non-diabetic	82	97.6	65	90.3	234	95.9	0.434
	Type1	0	0	1	1.4	2	0.8	
	Type2	0	0	1	1.4	1	0.4	
	Gestational diabetes	2	2.4	5	6.9	7	2.9	
Diabetes treated by	Diet	2	2.4	6	8.3	7	2.9	
	Diet and insulin	0	0	1	1.4	3	1.2	
	Not applicable	66	78.6	54	75.0	160	65.6	
Did the mother have liver disease?	No	84	100.0	71	98.6	243	99.6	0.448
	Yes			1	1.4	1	0.4	
Did the mother have a renal impairment?	No	84	100.0	72	100.0	243	99.6	0.726
	Yes	0	0	0	0	1	0.4	
Did the mother have uterine cancer?	No	84	100.0	72	100.0	244	100.0	
Did the mother have hypertension?	No	83	98.8	71	98.6	240	98.4	0.955
	Yes	1	1.2	1	1.4	4	1.6	
Did the mother have malaria?	No	84	100.0	72	100.0	244	100.0	
Did the mother have any other disease? (others)	No	81	96.4	69	95.8	234	96.3	0.979
	Yes	3	3.6	3	4.2	9	3.7	

Table 3 shows the maternal outcomes during pregnancy and delivery in relation to the anemia status of the mother. Pregnancy-induced hypertension was present among 5.6% (n = 4) of mothers with mild anemia, 1.2% (n = 3) of normal mothers, and none of the mothers with moderate anemia, the difference between the groups being statistically significant (p=0.019). Antepartum hemorrhage was seen in 6% (n=5) mothers with moderate anemia and 4% (n=1) normal mothers, while no mother with mild anemia had it, the difference between the groups being statistically significant (p=0.001). Postpartum hemorrhage as a complication of delivery was found more in moderately anemic mothers (14.3%; n=12) compared to those with mild anemia (6.9%; n=5) and normal mothers (3.3%; n = 8), with a statistically significant difference between them (p=0.002). None of the mothers with mild anemia took blood transfusions during their pregnancy, while 6% (n=5) of moderately anemic and 1.2% (n=3) normal mothers took blood transfusions (p=0.012). Emergency cesarean section was performed in 33.3% (n=24) of mildly anemic mothers, 31% (n=26) of moderately anemic mothers, and 19.3% (n=47) of normal mothers (p=0.017).

**Table 3:** Maternal outcomes during pregnancy and delivery by anemia status *Significant p-value

Maternal outcomes during pregnancy and delivery		Maternal hemoglobin g/dl						P-value
		Moderate		Mild		Normal		
		7–9.9 g/dl		10–10.9 g/dl		≥11 g/dl		
		Number	%	Number	%	Number	%	
Did the mother have Pregnancy induced hypertension?	No	84	100.0	68	94.4	241	98.8	0.019*
	Yes	0	0	4	5.6	3	1.2	
Did the mother have Preeclampsia?	No	82	97.6	71	98.6	242	99.2	0.536
	Yes	2	2.4	1	1.4	2	0.8	
Did the mother have an antepartum hemorrhage?	No	79	94.0	72	100.0	243	99.6	0.001*
	Yes	5	6.0	0	0	1	0.4	
Did the mother have intrauterine growth retardation?	No	82	97.6	71	98.6	242	99.2	0.536
	Yes	2	2.4	1	1.4	2	0.8	
Amniotic fluid index	<2cm	3	3.6	0	0	3	1.2	0.099
	>8cm	14	16.7	11	15.3	23	9.4	
	2-8cm	67	79.8	61	84.7	218	89.3	
Did the mother have shoulder dystocia delivery complication?	No	84	100.0	72	100.0	244	100.0	
Did the mother have fetal trauma delivery complication?	No	84	100.0	72	100.0	244	100.0	
Did the mother have second/third perinea degree tear delivery complication?	No	82	97.6	71	98.6	238	97.5	0.862
	Yes	2	2.4	1	1.4	6	2.5	
Did the mother have postpartum hemorrhage delivery complications?	No	72	85.7	67	93.1	236	96.7	0.002*
	Yes	12	14.3	5	6.9	8	3.3	
Did the mother take a blood transfusion during pregnancy?	No	79	94.0	72	100.0	241	98.8	0.012*
	Yes	5	6.0			3	1.2	
Mode of delivery	Spontaneous vaginal delivery	50	59.5	37	51.4	172	70.5	0.017*
	Elective cesarean section	8	9.5	10	13.9	25	10.2	
	Instrumental delivery			1	1.4			
	Emergency cesarean section	26	31.0	24	33.3	47	19.3	
Maternal admission to intensive care unit for any reason	No	80	95.2	70	97.2	242	99.2	0.073
	Yes	4	4.8	2	2.8	2	0.8	

With regard to the neonatal outcomes in relation to maternal anemia status, congenital malformations were diagnosed in 9.7% (n=7) cases where the mother was mildly anemic, 9.5% (n=8) moderately anemic, and 2% (n=5) of normal mothers, with a statistically significant difference between the groups (p=0.003). Admission to the neonatal intensive care unit (NICU) was higher among the neonates born to mothers with mild anemia, 12.5% (n =9), as compared to mothers with moderate anemia, 11.9% (n =10), and normal mothers, 1.6% (n =4), with a statistically significant difference between the groups (p<0.001) (Table 4).

**Table 4:** Neonatal outcomes by anemia status *Significant p-value

Neonatal outcomes		Maternal hemoglobin g/dl						P-value
		Moderate		Mild		Normal		
		7–9.9 g/dl		10–10.9 g/dl		≥11 g/dl		
		Number	%	Number	%	Number	%	
Living status	Alive born	84	100.0	72	100.0	244	100.0	
Newborn gender	Female	36	42.9	32	44.4	117	48.0	0.681
	Male	48	57.1	40	0.0	127	52.0	
Congenital malformation diagnosed	No	76	90.5	65	90.3	239	98.0	0.003*
	Yes	8	9.5	7	9.7	5	2.0	
Cardiac anomaly	No	81	96.4	71	98.6	240	98.4	0.507
	Yes	3	3.6	1	1.4	4	1.6	
Renal anomaly	No	82	97.6	71	98.6	244	100.0	0.073
	Yes	2	2.4	1	1.4	243	99.6	
Central nervous system anomaly	No	84	100.0	72	100.0	1	0.4	
Musculoskeletal anomaly	No	84	100.0	69	95.8	242	99.2	0.726
	Yes			3	4.2	2	0.8	
Neonatal admission to the neonatal intensive care unit	No	74	88.1	63	87.5	240	98.4	<0.001*
	Yes	10	11.9	9	12.5	4	1.6	
Birth weight	Less than 2.5 (low birthweight)	19	22.62	8	11.11	25	10.25	0.062
	2.5-5.5	64	76.19	63	87.50	214	87.70	
	More than 5.5	1	1.19	1	1.39	5	2.05	

A univariate logistic regression model presented in Table 5 shows a statistically significant association between anemia during pregnancy and postpartum hemorrhage as a delivery complication (odds ratio [OR] = 3.61; confidence interval [CI] 1.52-8.58; p=0.004); diagnosis of congenital malformation (OR = 5.09; CI 1.81-14.29; p=0.002); neonatal admission to the neonatal intensive care unit (OR = 8.32; CI 2.77-24.96; p=0.001); and low birth weight (OR = 1.833; CI 1.021-3.294; p=0.042).

**Table 5:** Univariate logistic regression for the associated risk factors with anemia in pregnancy *Significant P-value **Used as a reference

	Odds ratio	95% CI		P value
		Lower	Upper	
Diabetes	1.43	0.57	3.61	0.446
Liver disease	1.57	0.10	25.25	0.751
Hypertension	1.28	0.23	7.09	0.775
Did the mother have pregnancy-induced hypertension?	2.11	0.47	9.58	0.331
Did the mother have preeclampsia?	2.37	0.39	14.36	0.347
Did the mother have an antepartum hemorrhage?	8.05	0.93	69.54	0.058
Did the mother have intrauterine growth retardation?	2.37	0.39	14.36	0.347
Amniotic fluid index				
<2 cm**	1.00			
>8 cm	1.09	0.20	5.94	0.923
2–8 cm	0.59	0.12	2.95	0.518
Did the mother have postpartum hemorrhage delivery complications?	3.61	1.52	8.58	0.004*
Did the mother take a blood transfusion during pregnancy?	2.66	0.63	11.29	0.185
Newborn gender				
Male	1.19	0.80	1.79	0.394
Female**	1.00			
Congenital of malformation diagnosed	5.09	1.81	14.29	0.002*
Cardiac anomaly	1.58	0.39	6.41	0.523
Musculoskeletal anomaly	2.37	0.39	14.36	0.347
Neonatal admission to the neonatal intensive care unit	8.32	2.77	24.96	0.001*
Birth weight				
Less than 2.5 kg (low birthweight)	1.833	1.021	3.294	0.042*
≥2.5 kg**	1.00			

## Discussion

In the current study, the overall prevalence of anemia was found to be 39%, higher than the national prevalence estimated by the World Bank at 27.3% [4]. We presume this could be owing to the high parity of the study sample and in view that high parity has been indicated in the etiology of anemia in pregnancy [13].

We found a statistically significant relationship between anemia in pregnancy and postpartum hemorrhage and also reported the occurrence of postpartum hemorrhage to be higher in moderately anemic mothers compared to those with mild anemia and normal mothers; this result is in line with the findings of Nair et al. who reported that women with severe anemia had nine times higher odds of postpartum hemorrhage when compared to normal women and those with mild anemia (adjusted OR (aOR) 9.45; 95% CI 2.62 to 34.05) [14]. This is noteworthy since some previous studies have reported no association between maternal anemia and postpartum hemorrhage [15], and no difference in risk of postpartum hemorrhage between severe and non-severe anemia [16].

In relation to neonatal outcomes, our study demonstrated a significant association of maternal anemia with the development of congenital malformations; this is in contrast with some previous studies where no statistical difference was seen between anemic and non-anemic groups [17]. Moreover, our results suggest a significant relationship between maternal anemia and low birth weight; this is comparable with the findings of Parks et al., where severe maternal anemia was associated with low birth weight [18]. Furthermore, our study is consistent with the findings of Lin et al. who reported higher NICU admissions in mothers with anemia [19]. The increase in NICU admissions can be justified in view of higher rates of low birth weight and congenital malformations in neonate.

The limitations of the present study are that it only included women attending a single hospital in the central region of Saudi Arabia. Moreover, we did not measure ferritin levels, and since ferritin levels determine the type of anemia, it would be recommended to do so in future studies.

Our findings underscore the need to continue interventional programs to prevent and treat anemia, including preconception counseling and antenatal care. In conclusion, the study highlights the association of maternal anemia with adverse events in mothers, such as postpartum hemorrhage. Among neonates, congenital malformations, low birth weight, and higher admissions to the NICU have been reported.

## Conclusions

In conclusion, the prevalence of anemia in pregnant women (39%) shows a constant problem in public health in Saudi Arabia. Anemia in pregnancy has adverse effects on maternal and fetal outcomes. The highest prevalence of maternal outcomes was emergency cesarean section in mildly anemic mothers (33.3%) and postpartum hemorrhage in moderately anemic mothers (14.3%).

The significant fetal outcomes that were associated with maternal anemia are low birth weight in moderately anemic mothers (22.7%), NICU admissions in mildly anemic mothers (12.5%), and the development of congenital malformations (9.7%) in mildly anemic mothers, which indicates an important need to identify the root causes and risk factors that lead to anemia in pregnancy. It is recommended to continue the interventional programs to prevent and treat anemia, including preconception counseling and antenatal care.
